# FANCD2 limits acetaldehyde‐induced genomic instability during DNA replication in esophageal keratinocytes

**DOI:** 10.1002/1878-0261.13072

**Published:** 2021-08-08

**Authors:** Jasmine D. Peake, Chiaki Noguchi, Baicheng Lin, Amber Theriault, Margaret O'Connor, Shivani Sheth, Koji Tanaka, Hiroshi Nakagawa, Eishi Noguchi

**Affiliations:** ^1^ Program in Molecular and Cellular Biology and Genetics Graduate School of Biomedical Sciences and Professional Studies Drexel University College of Medicine Philadelphia PA USA; ^2^ Department of Biochemistry and Molecular Biology Drexel University College of Medicine Philadelphia PA USA; ^3^ Program in Cancer Biology Graduate School of Biomedical Sciences and Professional Studies Drexel University College of Medicine Philadelphia PA USA; ^4^ Gastroenterology Division Department of Medicine University of Pennsylvania Perelman School of Medicine Philadelphia PA USA; ^5^ Division of Digestive and Liver Diseases Department of Medicine Columbia University Herbert Irving Comprehensive Cancer Center New York NY USA; ^6^ Present address: Department of Gastroenterological Surgery Graduate School of Medicine Osaka University Suita Japan

**Keywords:** acetaldehyde, alcohol, esophageal squamous cell carcinoma, FANCD2, Fanconi anemia, genomic instability

## Abstract

Individuals with Fanconi anemia (FA), a rare genetic bone marrow failure syndrome, have an increased risk of young‐onset head and neck squamous cell carcinomas (SCCs) and esophageal SCC. The FA DNA repair pathway is activated upon DNA damage induced by acetaldehyde, a chief alcohol metabolite and one of the major carcinogens in humans. However, the molecular basis of acetaldehyde‐induced genomic instability in SCCs of the head and neck and of the esophagus in FA remains elusive. Here, we report the effects of acetaldehyde on replication stress response in esophageal epithelial cells (keratinocytes). Acetaldehyde‐exposed esophageal keratinocytes displayed accumulation of DNA damage foci consisting of 53BP1 and BRCA1. At physiologically relevant concentrations, acetaldehyde activated the ATR‐Chk1 pathway, leading to S‐ and G2/M‐phase delay with accumulation of the FA complementation group D2 protein (FANCD2) at the sites of DNA synthesis, suggesting that acetaldehyde impedes replication fork progression. Consistently, depletion of the replication fork protection protein Timeless led to elevated DNA damage upon acetaldehyde exposure. Furthermore, FANCD2 depletion exacerbated replication abnormalities, elevated DNA damage, and led to apoptotic cell death, indicating that FANCD2 prevents acetaldehyde‐induced genomic instability in esophageal keratinocytes. These observations contribute to our understanding of the mechanisms that drive genomic instability in FA patients and alcohol‐related carcinogenesis, thereby providing a translational implication in the development of more effective therapies for SCCs.

## Graphical abstract

Esophageal squamous cell carcinoma (ESCC) is common in patients with Fanconi anemia (FA). Using esophageal keratinocytes, this study revealed that FANCD2, a central player of the FA DNA repair pathway, minimizes DNA replication stress in response to physiological levels of acetaldehyde, the primary metabolite of alcohol. This study suggests that FANCD2 prevents acetaldehyde‐induced genomic instability during replication to prevent ESCC.

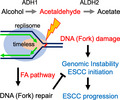

**Abbreviations**
BER: base excision repair
DPC: DNA–protein crosslink
EdU: 5‐ethynyl‐2′‐deoxyuridine
ESCC: esophageal squamous cell carcinoma
FA: Fanconi anemia
FANCD2: Fanconi anemia complementation group D2
FPC: replication fork protection complex
HR: homologous recombination
HSCs: hematopoietic stem cells
HSPC: hematopoietic stem and progenitor cell
ICL: interstrand crosslink
*N* ^2^‐Ethylidene‐dG: *N* ^2^‐ethylidene‐2′‐deoxyguanosine
NER: nucleotide excision repair
NHEJ: nonhomologous end joining
PdG: 1,*N* ^2^‐propano‐2′‐deoxyguanosine
SCC: squamous cell carcinoma
shRNA: short hairpin RNA
siRNA: small interfering RNA
TLS: translesion synthesis

## 1 Introduction

Fanconi anemia (FA) is characterized by bone marrow failure and predisposition to malignancies including leukemia and young‐onset squamous cell carcinomas (SCCs) of the head and neck and of the esophagus [1]. With improved survival for leukemia patients as a result of bone marrow transplantation, SCCs are the life‐threatening issues in young adults with FA [2, 3]. FA patients have deficiency in the FA DNA repair pathway (FA pathway), which is designed to remove DNA interstrand crosslinks (ICLs) induced by DNA‐damaging agents [4, 5], including acetaldehyde, the primary metabolite of alcohol.

At least 22 genes are associated with the FA pathway. When the DNA replication machinery encounters an ICL, 13 FA proteins (FANCA, B, C, E, F, G, L, M, and T; FA‐associated proteins FAAP10, FAAP16, FAAP24, FAAP100) form the FA core complex at the replication fork [4, 5]. This core complex acts as the E3 ubiquitin ligase for FANCI‐FANCD2 complex, which, together with the ATR cell cycle checkpoint kinase, plays a critical role in coordinating downstream DNA repair processes. These repair processes include nucleotide excision repair (NER), translesion synthesis (TLS), and homologous recombination (HR), all of which are required to complete ICL repair in order to resume DNA replication and preserve genomic integrity [4, 5].

Dysfunctional FA gene products have been linked to acetaldehyde‐induced DNA damage and chromosomal aberrations [6, 7, 8]. FANCD2 is monoubiquitinated and thereby activated, in response to alcohol (ethanol) or acetaldehyde treatment in human cells [9, 10]. Acetaldehyde‐induced DNA damage has been implicated in developmental defects, bone marrow failure, and acute leukemia in alcohol‐treated FANCD2 knockout mice [11], indicating the importance of the FA pathway as a cytoprotective mechanism against acetaldehyde.

Acetaldehyde directly reacts with deoxyguanosine residues in DNA to form DNA adducts such as *N* ^2^‐ethylidene‐2′‐deoxyguanosine (*N* ^2^‐Ethylidene‐dG) and 1,*N* ^2^‐propano‐2′‐deoxyguanosine (PdG) [6]. Although these monoadducts themselves may cause problems during DNA transactions, PdG adducts can generate more toxic lesions including DNA ICLs and DNA–protein crosslinks (DPCs) [6]. ICLs and DPCs interfere with the DNA replication and repair processes, leading to genomic instability, which is a hallmark of cancer [6, 8, 12].

Previous studies used various human cells to demonstrate that acetaldehyde induces DNA damage and activates DNA repair mechanisms. For example, CHO cells deficient in HR and NER pathways experience chromosomal aberrations in response to acetaldehyde [13]. The same study also showed that mutations in BER or NHEJ also display mild genomic instability [13]. Such genomic instability is likely to stem from acetaldehyde‐induced DNA adducts, including ICLs, which activate the FA pathway. Consistently, CHO cells deficient for FA proteins such as FANCQ/XPF and FANCG display chromosome aberrations in response to acetaldehyde [14]. Furthermore, a number of studies reported roles of FA proteins in acetaldehyde‐induced DNA damage response in various cell lines and tissues, including chicken DT40 cells, lymphoblastoids, hematopoietic stem cells (HSCs), fibroblasts, and various cancer cells [10, 11, 15, 16, 17, 18, 19]. Although knowledge regarding how acetaldehyde affects genomic integrity has been accumulating, it is still largely elusive as to how acetaldehyde causes genomic instability and how the FA pathway protects against acetaldehyde‐induced DNA damage and carcinogenesis in esophageal cells. This is a significant knowledge gap, given that there is a strong genetic link among acetaldehyde accumulation, the FA pathway, and esophageal cancers [20].

In this report, we show that acetaldehyde promotes replication stress in human esophageal keratinocytes. At physiologically relevant concentrations, acetaldehyde treatment results in replicative DNA damage and activates the ATR‐Chk1‐dependent DNA damage response, resulting in the accumulation of cells at the S and G2/M phases. We also show that the FA pathway is activated in response to acetaldehyde and that the disruption of the FA pathway prevents efficient DNA repair and replication fork recovery after acetaldehyde treatment. These results serve as a stepping‐stone for future studies to develop effective prevention, early detection, and therapy of FA‐ and alcohol‐associated SCCs.

## 2 Materials and methods

### 2.1 Cell culture

Immortalized normal human keratinocyte cell lines EPC1‐hTERT, EPC2‐hTERT, and EPC3‐hTERT cells were cultured in keratinocyte serum‐free medium (Thermo Fisher Scientific, Waltham, MA, USA) supplemented with 50 µg·mL^−1^ bovine pituitary extract, 1 ng·mL^−1^ human recombinant epidermal growth factor, 100 U·mL^−1^ penicillin, and 100 µg·mL^−1^ streptomycin, as previously described [21, 22, 23, 24]. TE11 cells were cultured in DMEM supplemented with 10% FBS, penicillin, and streptomycin as described above. To generate 3‐dimensional (3D) organoids, 100 TE11 cells were plated in 5 µL of Matrigel basement membrane matrix (Corning, New York, NY, USA) in 96‐well plates and grown for 13 days, and organoid formation rate was determined as described in our previous studies [25, 26].

### 2.2 Acetaldehyde treatment

Acetaldehyde has a boiling point of 20.2 °C; thus, it is extremely volatile. For this reason, acetaldehyde solution was stored at −20 °C prior to treatment. Pipet tips were also kept at −20 °C before pipetting acetaldehyde. In order to minimize acetaldehyde evaporation, all reagents and equipment were kept on ice while preparing acetaldehyde‐containing medium. Acetaldehyde (Sigma‐Aldrich, St Louis, MO, USA, 402788) was serially diluted to reach 17.8 mm in growth medium, then added to cell/medium containing T12.5 or T25 flasks at an initial concentration of 0.25, 0.5, 1, or 2 mm, and sealed for the duration of treatment. WST‐8 cell viability assay and acetaldehyde treatment of 3D‐organoid cultures were performed in a 96‐well plate that was sealed with an adhesive storage foil (USA Scientific, Ocala, FL, USA). For WST‐8 cell viability assay, cells were grown in 96‐well plates and transfected with small interfering RNAs (siRNAs). Two days after transfection, cells were treated with acetaldehyde for 72 h and subjected to WST‐8 viability assay following the instructions by the supplier (ab228554; Abcam, Cambridge, MA, USA).

Acetaldehyde evaporation is unavoidable; the initial concentrations of acetaldehyde in growth medium are indicated throughout this study. The acetaldehyde treatment scheme is shown in Fig. S1.

### 2.3 Cell cycle analysis

For cell cycle analysis, EPC2‐hTERT cells were plated in T12.5 flasks, grown for 48 h, and treated with acetaldehyde for 24 h. When cell cycle synchronization was required, EPC2‐hTERT cells were synchronized at the G1/S boundary with 200 µm l‐mimosine (Sigma‐Aldrich; M0253) for 24 h prior to acetaldehyde treatment. Cells were then collected, washed with 1× PBS, and fixed in 70% ethanol at −20 °C. Fixed cells were precipitated, rehydrated in 1× PBS, denatured with 2 N HCl, and neutralized with 0.1 m Na_2_B_4_O_7_ pH 8.5. Cells were precipitated again and incubated in the presence of 3.8 mm sodium citrate, 10 µg·mL^−1^ propidium iodide, and 500 µg·mL^−1^ RNaseA. Finally, cells were processed for cell cycle analysis using a Guava EasyCyte Plus flow cytometer (Millipore, Billerica, MA, USA) or a BD LSRFortessa cell analyzer (BD Biosciences, Franklin Lakes, NJ, USA).

### 2.4 RNAi

Constitutive short hairpin RNA (shRNA)‐mediated gene silencing was performed using the lentiviral vector pLKO.1‐PURO encoding shRNA sequences for scrambled control (Addgene plasmid #1864, described previously [27]), FANCD2 (mature antisense: AATCCTCCAATCTAATAGACG, GE Dharmacon TRCN0000082838), and TIMELESS (mature antisense: AATGCAATGGTTAGTGTGGGC, GE Dharmacon TRCN0000157211, described previously [27]). Doxycycline‐inducible shRNA‐mediated gene silencing was performed using the pTRIPZ inducible lentiviral vector encoding shRNA sequences for nonsilencing control (GE Dharmacon RHS4743) and FANCD2 (mature antisense: TACCTCAAGTGTATCCATG, GE Dharmacon V2THS_139155). All shRNA‐expressing viral particles were produced as described [27]. Cells were infected with lentiviral particles under centrifugal force at 1000 ***g*** for 1 h at 32 °C followed by overnight incubation at 37 °C. To select cells stably depleted of FANCD2, cells were further incubated without lentivirus for 24 h and selected with 0.5 µg·mL^−1^ puromycin for 4–5 days. FANCD2 depletion was routinely monitored via western blotting.

Transfection of siRNA duplexes was performed by using Mission**^®^** siRNA Transfection Reagent (S1452; Sigma‐Aldrich, St. Louis, MO, USA). siRNAs were purchased from Sigma‐Aldrich: Mission**^®^** Universal Negative Control #1 (SIC001), FANCD2‐siRNA‐#1 (SASI_Hs01_00137854), and FANCD2‐siRNA‐#2 (SASI_Hs02_00307032).

### 2.5 Antibodies

Antibody to Phospho‐S345‐Chk1 (#2348) and Phospho‐S10‐Histone H3 (#53348) was purchased from Cell Signaling Technology (Danvers, MA, USA); BRCA1 (sc‐6954), Cyclin A (sc‐2712682), Chk1 (sc‐8408), and ATR (sc‐1887) from Santa Cruz (Dallas, TX, USA); p53 (Ab‐6, Clone DO‐1, MS‐187‐P0) from Thermo Fisher Scientific; 53BP1 (MAB3802) and γH2AX S139 (05‐636) from MilliporeSigma (Burlington, MA, USA); and FANCD2 (NB100‐182) from Novus Biologicals (Centennial, CO, USA). The polyclonal anti‐Timeless antibody was generated previously [28].

### 2.6 Western blotting

Cells were grown and treated with up to 1 mm acetaldehyde for 24–72 h. Cells were then collected and lysed with RIPA1 buffer [50 mm Tris pH 7.2, 150 mm NaCl, 0.1% SDS, 0.5% DOC, 1% NP‐40, 2 mm EDTA, 50 mm NaF, 2 mm PMSF, 1X HALT protease inhibitor (Thermo Fisher Scientific)] and centrifuged at 16 100 ***g*** in an Eppendorf microcentrifuge 5414R for 10 min at 4 °C. After the supernatant was collected, protein concentrations were determined using the Pierce BCA protein assay kit (Thermo Fisher Scientific). Samples were then boiled in the presence of bromophenol blue and 5% beta‐mercaptoethanol for 5 min, after which protein concentrations were equalized. Protein lysates were run on SDS/polyacrylamide gels, and proteins were transferred onto PVDF membranes and exposed to the indicated antibodies for probing. To enhance the mobility shift associated with Chk1 phosphorylation, SDS/polyacrylamide gels with acrylamide : bisacrylamide in 150 : 1 ratio were used, while SDS/polyacrylamide gels with acrylamide : bisacrylamide in 29.2 : 0.8 ratio were used for all other western blotting analyses.

### 2.7 Immunofluorescence microscopy and abnormal nuclear structures

Cells were grown on coverslips in T25 flasks and treated with up to 1 mm acetaldehyde for 24 h. Cells were treated with cytoskeleton striping buffer (25 mm HEPES pH 7.5, 50 mm NaCl, 1 mm EDTA, 3 mm MgCl_2_, 300 mm Glucose, 0.5% Triton X‐100), fixed (3% Paraformaldehyde, 2% Sucrose in PBS), permeabilized (0.5% Triton X‐100 in PBS), blocked (5% FBS in PBS), and immunostained with the indicated primary antibody followed by incubation with the corresponding fluorophore‐conjugated secondary antibody as described previously [29]. Coverslips were then rinsed and mounted using ProLong Gold Antifade Mountant with DAPI (Thermo Fisher Scientific) for microscopic analysis using an Olympus PROVIS AX70 microscope equipped with a Retiga EXi camera (QImaging, Surrey, BC, Canada). Images were obtained with ivision software (BioVision Technologies, Exton, PA, USA) and analyzed with imagej software (National Institutes of Health, Bethesda, MD, USA). At least 50 cells were scored for each experiment. To evaluate abnormal nuclear structures, cells were fixed and stained with DAPI, and images were analyzed as described above.

### 2.8 EdU fluorescent microscopy

Cells were plated on coverslips in T25 flasks, grown for 24 h, and treated with acetaldehyde for 24 h. Cells were then released into growth medium containing 10 µm 5‐ethynyl‐2′‐deoxyuridine (EdU) for 30 min, fixed, and permeabilized as described above. For detection of EdU, Click‐IT EdU Alexa Fluor 488 Imaging (Thermo Fisher Scientific; C10337) was used following manufacturer's instruction. Microscopic analysis was performed as described above.

## 3 Results

### 3.1 Acetaldehyde causes ATR‐Chk1‐dependent G2/M delay in esophageal keratinocytes

**Fig. 1:**
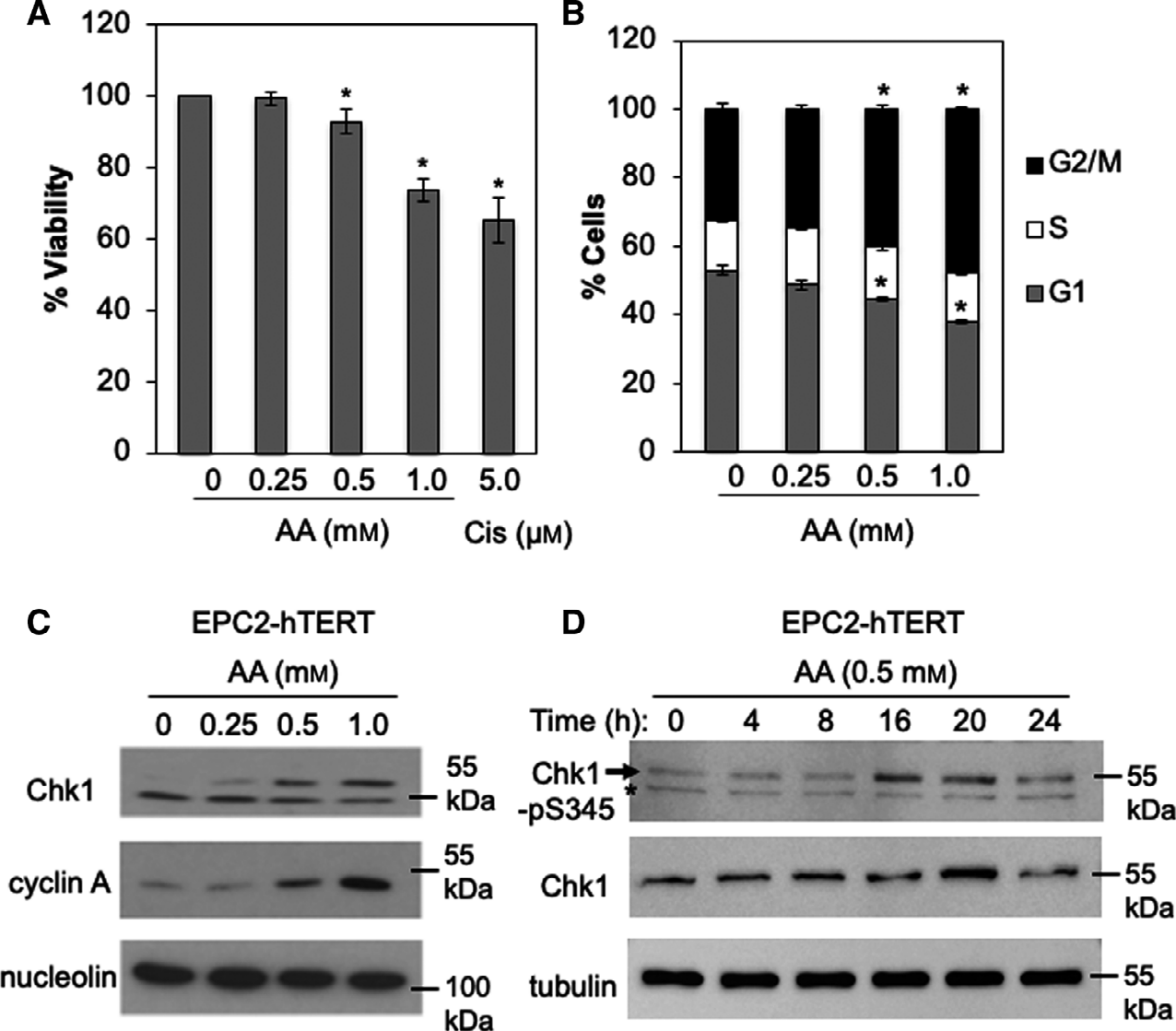
Acetaldehyde activates DNA damage response and causes G2/M arrest in EPC2‐hTERT cells. (A) EPC2‐hTERT cells were treated with up to 1 mm acetaldehyde (AA) or a crosslinking control (5 µm cisplatin, Cis) for 48 h. Cells were then treated with trypan blue to assess cell viability. Two hundred cells were counted for each condition. *n* = 3. Student's *t*‐test, *P < 0.05. Error bars correspond to standard errors of the mean (SEM). (B) EPC2‐hTERT cells were treated with up to 1 mm AA for 24 h. Cells were then collected and stained with propidium Iodide for flow cytometry analysis using a Guava EasyCyte Plus flow cytometer. Ten thousand cells were counted for each condition. *n* = 3. Student's *t*‐test, *P < 0.05. Error bars correspond to SEM. (C) EPC2‐hTERT cells were treated with up to 1 mm AA for 24 h. Cell lysates were prepared and probed for the indicated proteins. Arrows indicate phosphorylated Chk1. Nucleolin was used as a loading control. (D) EPC2‐hTERT cells were treated with 0.5 mm AA for the indicated times and processed for immunoblot analyses with the indicated antibodies. Arrows indicate Chk1 phosphorylated at S345. Asterisk shows nonspecific bands. Representative results of repeat experiments are shown.

To understand the effects of acetaldehyde on the esophagus, we sought to determine physiologically relevant concentrations of acetaldehyde to treat nontransformed esophageal keratinocytes (EPC1/2/3‐hTERT cells [22]). Studies reported that acetaldehyde concentrations of beer and red wine are 0.14 and 0.25 mm, respectively [30]. Another study found that short‐term alcoholic beverage exposure in the mouth leads to a salivary acetaldehyde concentration of up to 1 mm [31]. Therefore, to recapitulate physiological conditions of acetaldehyde exposure in the esophagus after alcohol drinking, we treated cells with increasing acetaldehyde concentrations up to 1 mm for 24–72 h. In addition, acetaldehyde is highly volatile, and its evaporation during experiments is unavoidable. In order to minimize acetaldehyde evaporation, acetaldehyde treatment was performed in sealed flasks or plates in most experimental settings (Fig. S1). As shown in Fig. 1A, EPC2‐hTERT cells displayed a significant decrease in cell viability in response to acetaldehyde. A similar effect was observed when cells were treated with cisplatin, a chemotherapeutic agent known to generate ICLs and cause cell death [32] (Fig. 1A).

We also used the esophageal squamous cell carcinoma (ESCC) cell line TE11 [33]. TE11 cells were resistant up to 1 mm of acetaldehyde (Fig. S2A). Therefore, we pre‐incubated TE11 cells with disulfiram, a potent inhibitor of aldehyde dehydrogenase 2 (ALDH2) [34]. Under this condition, TE11 cells showed significant sensitivity to acetaldehyde (Fig. S2B).

Physiological levels of acetaldehyde can lead to *N* ^2^‐Ethylidene‐dG DNA adduct formation in cell culture [35]. It is possible that such adducts also inhibit DNA synthesis, leading to activation of cell cycle checkpoints that inhibit progression of the cell cycle. To test this possibility, we treated cells with acetaldehyde for 24 h and processed the cells for cell cycle analysis. Flow cytometry analysis revealed that cells accumulated at the G2/M phase in the presence of acetaldehyde (Fig. 1B). Consistently, the level of cyclin A, a protein required during S and G2 phases, was elevated after acetaldehyde exposure in EPC2‐hTERT cells (Fig. 1C). Replication stress activates the ATR kinase, leading to phosphorylation, thus activation of Chk1, a master checkpoint kinase required for G2/M arrest [36]. Importantly, Chk1 is highly phosphorylated in response to acetaldehyde, which is represented by slower‐migrating species (Fig. 1C). Acetaldehyde‐induced Chk1 phosphorylation was also confirmed by probing Chk1 phosphorylated at S345 in EPC3‐hTERT cells (Fig. S3A). Phosphorylation of Chk1 on S345 was further evaluated in a time‐course experiment where EPC2‐hTERT cells were treated with 0.5 mm acetaldehyde. Chk1 phosphorylation started increase at 16 h and stayed high until 20 h (Fig. 1D). Similar results were also obtained using another esophageal keratinocyte cell line EPC1‐hTERT (Fig. S3B). Furthermore, we also observed phosphorylation of histone H3 on S10, a marker of G2/M and mitotic arrest when cells were treated with 1 mm acetaldehyde (Fig. S3A). Thus, our results suggest that acetaldehyde induces Chk1‐dependent G2/M arrest in esophageal keratinocytes, likely due to accumulation of DNA damage.

### 3.2 Acetaldehyde induces DNA damage, leading to apoptosis in esophageal keratinocytes

**Fig. 2:**
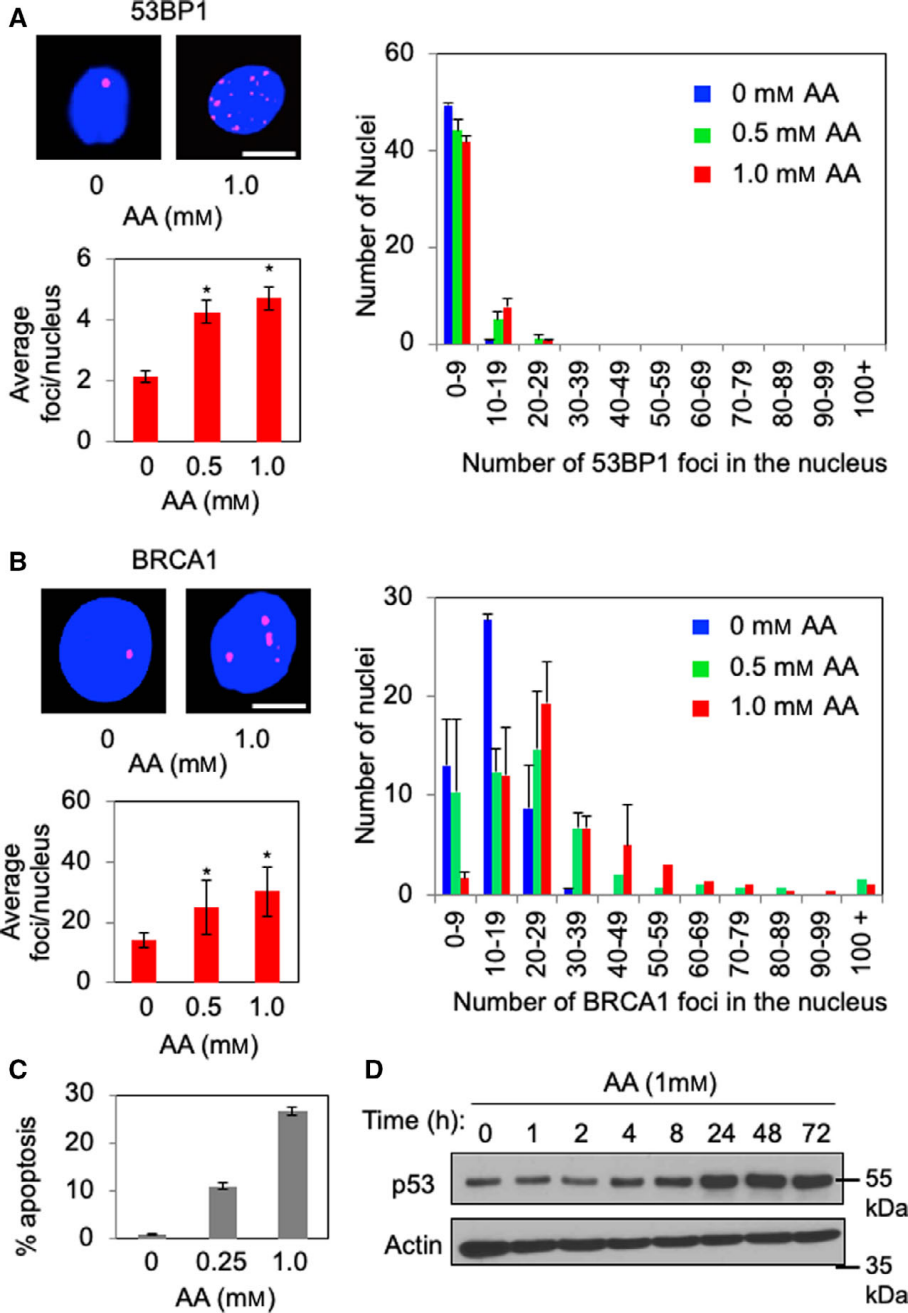
Acetaldehyde treatment promotes the formation DNA damage foci and leads to apoptotic cell death. (A, B) EPC2‐hTERT cells were treated with up to 1 mm acetaldehyde (AA) for 24 h. Cells were then fixed and processed for immunofluorescence to detect the localization of the indicated protein. Representative images of DNA damage foci are shown. Scale bars, 10 µm. Quantification of DNA damage foci is presented as average number of DNA damage foci per nucleus. At least 50 cells were counted for each replicate. *n* = 3. Mann–Whitney *U*‐test, *P < 0.05. Error bars represent SEM. In addition, the number of DNA damage foci in each nucleus was counted and scored within a category of foci number per nucleus. (C) EPC2‐hTERT cells were treated with up to 1 mm AA for 24 h and stained with Annexin V and propidium iodide to assess apoptotic cells by flow cytometry. Error bars correspond to SEM obtained from three independent experiments. (D) EPC2‐hTERT cells were treated with 1 mm AA for the indicated times. Cell lysates were prepared and probed for p53. Actin was used as a loading control. Representative results of repeat experiments are shown.

To investigate the effect of acetaldehyde on DNA damage response, we determined the levels of DNA damage in acetaldehyde‐treated esophageal cells. For this purpose, we monitored formation of 53BP1 and BRCA1 DNA damage foci [37] via immunofluorescence microscopy (IF) in EPC2‐hTERT esophageal keratinocytes that were treated with up to 1 mm of acetaldehyde. Importantly, acetaldehyde treatment resulted in accumulation of 53BP1 and BRCA1 foci, in a dose‐dependent manner (Fig. 2). As shown in Fig. 2A, there was a significant increase in the average number of 53BP1 foci per nucleus upon acetaldehyde treatment (Fig. 2A, left graph). We also determined the distribution of 53BP1 foci numbers in individual nuclei by categorizing each nucleus based on the number of 53BP1 foci it contained. This analysis further confirmed that acetaldehyde induces elevated levels of DNA damage represented by 53BP1 foci formation (Fig. 2A, right graph). Similarly, the number of nuclear BRCA1 foci also significantly increased in response to acetaldehyde (Fig. 2B). In addition, TE11 ESCC cells also displayed elevated levels of 53BP1 foci formation especially when cells were treated with the ALDH2 inhibitor disulfiram (Fig. S2C), indicating that acetaldehyde treatment induces DNA damage in esophageal cells.

It is possible that such DNA damage triggers apoptotic cell death. To test this possibility, we performed␣Annexin V/propidium iodide double staining of acetaldehyde‐treated EPC2‐hTERT cells. Indeed, acetaldehyde induced significant levels of apoptosis in these cells (Fig. 2C). Furthermore, p53, which is involved in activation of apoptosis in response to DNA damage, was elevated in response to acetaldehyde (Fig. 2D). Thus, acetaldehyde is a genotoxic agent in esophageal keratinocytes.

### 3.3 Acetaldehyde treatment causes activation of the FA pathway in esophageal keratinocytes

**Fig. 3:**
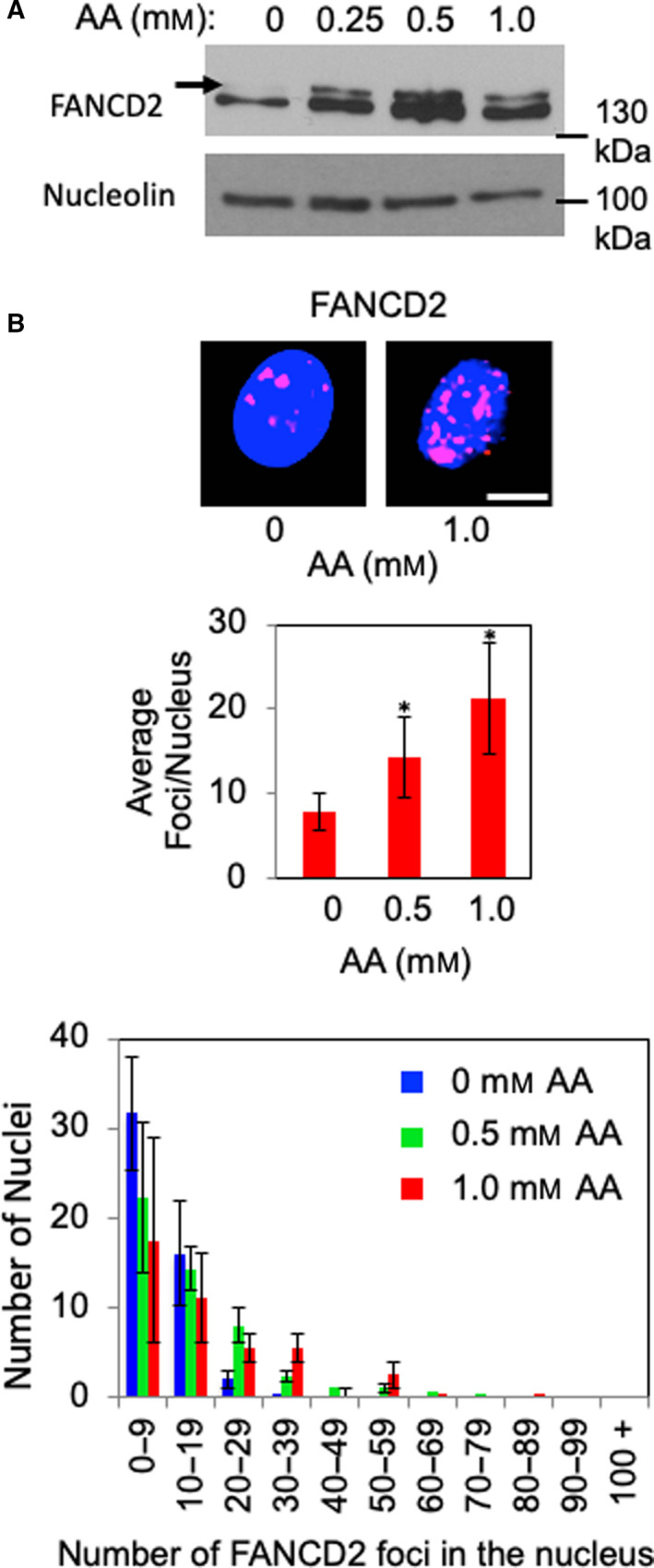
Acetaldehyde activates the FA pathway in EPC2‐hTERT cells. (A) EPC2‐hTERT cells were treated with up to 1 mm acetaldehyde (AA) for 24 h. Cell lysates were prepared and probed for the indicated proteins. Arrows indicate monoubiquitinated FANCD2. Nucleolin was used as a loading control. Representative results of repeat experiments are shown. Scale bar, 10 µm. (B) DNA damage foci analysis and statistical analysis were performed as described in Fig. 2A, except that FANCD2 localization was monitored. At least 50 cells were counted for each replicate. *n* = 3. Mann–Whitney *U*‐test, *P < 0.05. Error bars represent SEM.

Acetaldehyde is known to induce ICLs, which are repaired via the FA pathway. Therefore, we investigated the roles of the FA pathway in EPC2‐hTERT cells after acetaldehyde treatment. For this purpose, we monitored monoubiquitination of FANCD2, which␣is required for activating the FA pathway [38]. Indeed, FANCD2 was monoubiquitinated in response to acetaldehyde treatment in EPC2‐hTERT cells (Fig. 3A).

FANCD2 is known to form DNA damage foci when activated [38, 39]; therefore, we also monitored FANCD2 localization in response to acetaldehyde in EPC2‐hTERT cells. As shown in Fig. 3B, acetaldehyde induced a significant accumulation of FANCD2 foci formation in a dose‐dependent manner (Fig. 3B). Once ubiquitinated, FANCD2 recruits proteins involved in DNA repair pathways, including NER, TLS, and HR [4, 5]. Consistently, we also observed BRCA1 (also known as FANCS, HR factor) foci formation in response to acetaldehyde (Fig. 2B). Thus, we concluded that the FA pathway is activated in esophageal keratinocytes in response to acetaldehyde.

### 3.4 Acetaldehyde causes replication stress in esophageal keratinocytes

The aforementioned results prompted us to investigate how and where acetaldehyde‐induced DNA damage occurs. Considering that acetaldehyde promotes activation of the Chk1‐dependent checkpoint, responsible for managing DNA damage during S phase, we hypothesized that acetaldehyde incudes DNA damage during DNA replication.

**Fig. 4:**
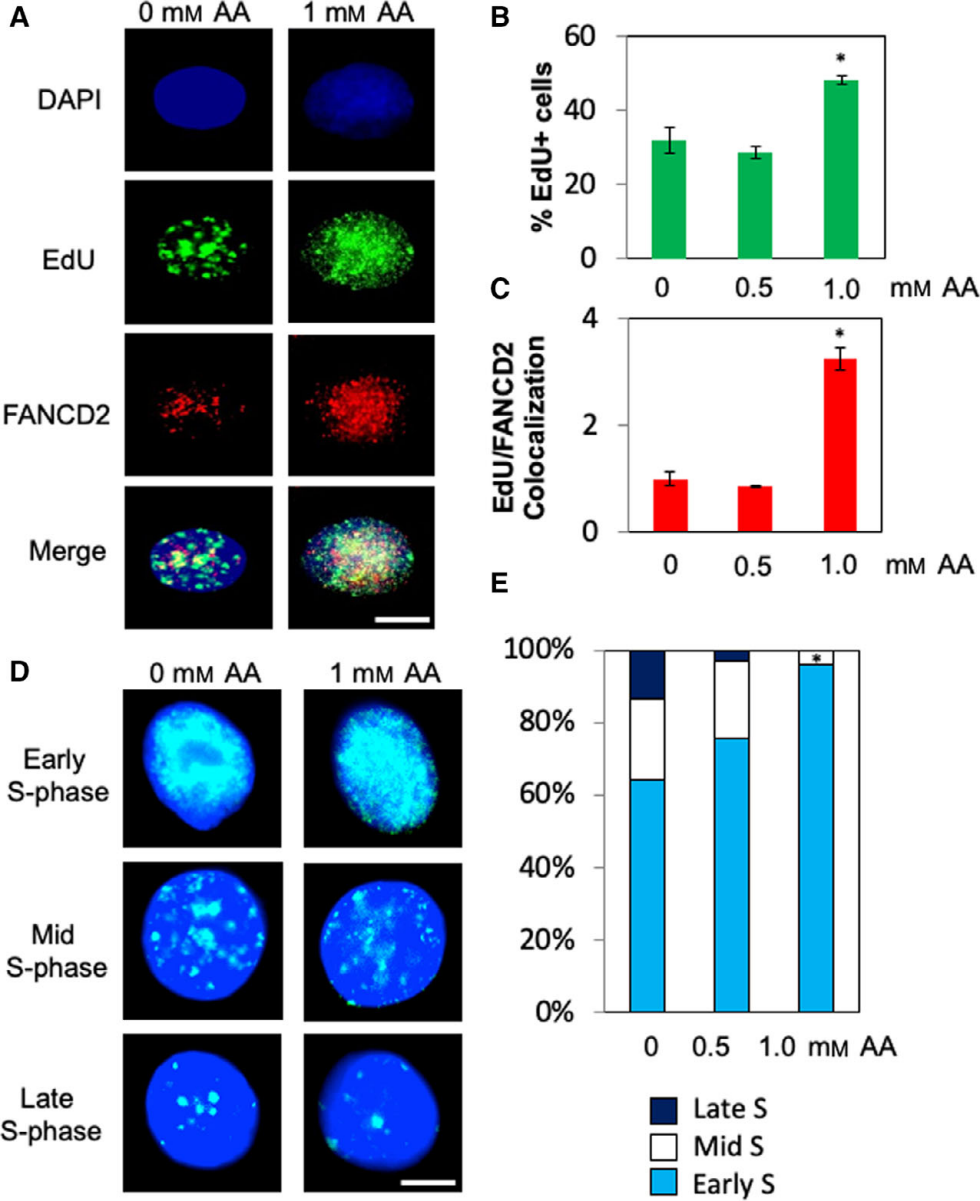
Acetaldehyde causes replicative DNA damage in esophageal keratinocytes. (A) EPC2‐hTERT cells were treated with up to 1 mm acetaldehyde (AA) for 24 h. Cells were then incubated in EdU for 30 min, fixed, and processed for EdU detection and immunofluorescence of FANCD2. Scale bar, 10 µm. (B, C) Quantification of EdU‐positive cells (in B) and FANCD2/EdU colocalization (in C). Colocalization analysis was performed using imagej. FANCD2 and EdU signals that were above the noise threshold of 250 were evaluated for colocalization. The acquired FANCD2 and EdU images were superimposed, and resulting yellow fluorescence that contained eligible FANCD2 and EdU signals was quantified. At least 50␣cells were counted for each experiment. *n* = 3. Mann–Whitney *U*‐test, *P < 0.05. Error bars represent SEM. (D) Representative images of EdU incorporation during early, mid‐, and late S phase. Scale bar, 10 µm. (E) Quantification of cells in early, mid‐, and late S phase. *n* = 3. Mann–Whitney *U*‐test, *P < 0.05.

To test this hypothesis, we incubated EPC2‐hTERT cells with EdU for 30 min after acetaldehyde treatment to allow cells to incorporate EdU into newly replicated DNA, marking sites of DNA synthesis. Interestingly, we observed an increase in percentage of EdU‐positive cells in response to acetaldehyde (Fig. 4A,B), suggesting that acetaldehyde treatment delays S‐phase progression, which is consistent with cyclin A accumulation observed in Fig. 1C. Importantly, there was a significant increase in colocalization of FANCD2 and EdU signals (Fig. 4C), indicating that FANCD2 forms DNA damage foci at the site of DNA synthesis. Thus, our results suggest that acetaldehyde induces DNA replication stress.

Previous studies established changes in the pattern of replication foci as cells replicate DNA in S phase. Replication foci are smaller and scattered throughout the nucleus during early S phase. During mid‐ or late S phase, these foci become larger, while the number of replication foci per nucleus decreases [40, 41]. Consistently, we also found that such pattern changes in replication foci labeled with EdU (Fig. 4D). Surprisingly, acetaldehyde treatment resulted in increased early S‐phase cells, as determined by their replication foci pattern (Fig. 4D,E). This result suggests that acetaldehyde‐induced DNA damage compromises S‐phase progression, leading to enrichment of cells in early S phase.

**Fig. 5:**
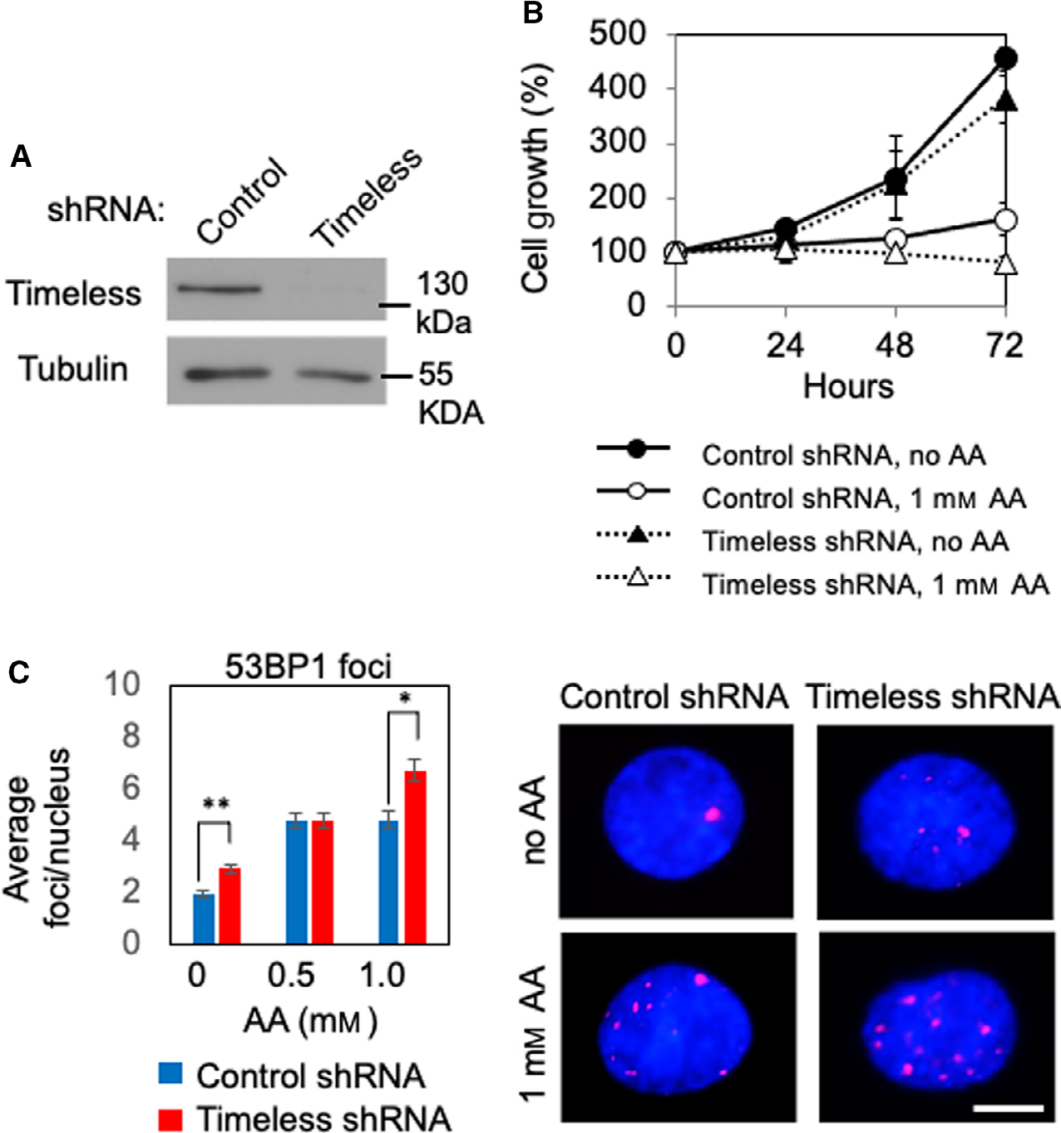
The fork protection protein Timeless limits acetaldehyde‐induced DNA damage. (A) EPC2‐hTERT cells were infected by lentivirus expressing the indicated shRNA and selected in the presence of puromycin. Western analysis demonstrates that Timeless was efficiently downregulated. (B) Cells expressing the indicated shRNA were treated with or without 1 mm acetaldehyde (AA) for 24 h. Cells were returned to fresh medium, incubated for the indicated time, and counted in triplicate. Cell growth in percentage (%) was calculated against the number of cells at 0 h. Error bars indicate SEM. (C) EPC2‐hTERT cells were treated with up to 1 mm AA for 24 h. Cells were then fixed and processed for immunofluorescence microscopy to detect 53BP1 DNA damage foci. Quantification of 53BP1 DNA damage foci is presented as average number of foci per nucleus. At least 50 cells were counted for each replicate. *n* = 3. Mann–Whitney *U*‐test; *P < 0.0005 and **P < 0.00001. Error bars represent SEM. Representative images of 53BP1 foci are shown. Scale bar, 10 µm.

Acetaldehyde‐induced replication stress would stimulate cells to protect replication fork structures. Central to replication fork protection stands the replication fork protection complex (FPC), comprised of the Timeless and Tipin proteins [42]. Studies have demonstrated that the FPC prevents replication fork collapse in response to DNA damage [42]. In addition, we previously reported that Swi1, the *Schizosaccharomyces pombe* ortholog of Timeless, is involved in replication fork stabilization upon acetaldehyde [8]. In order to strengthen the argument that acetaldehyde induces DNA damage at the replication fork, we depleted Timeless from EPC2‐hTERT cells to determine recruitment of DNA repair factors (Fig. 5A). When compared to control cells, Timeless‐depleted cells had severe growth defects in the presence acetaldehyde (Fig. 5B). We also noted that there was a slight elevation in the level of 53BP1 foci in Timeless‐depleted cells in response to acetaldehyde when compared to control cells (Fig. 5C). Taken together, we have concluded that acetaldehyde‐induced DNA lesions cause replication stress, leading to DNA damage.

### 3.5 FANCD2 limits acetaldehyde‐induced DNA replication stress and DNA damage in esophageal cells

**Fig. 6:**
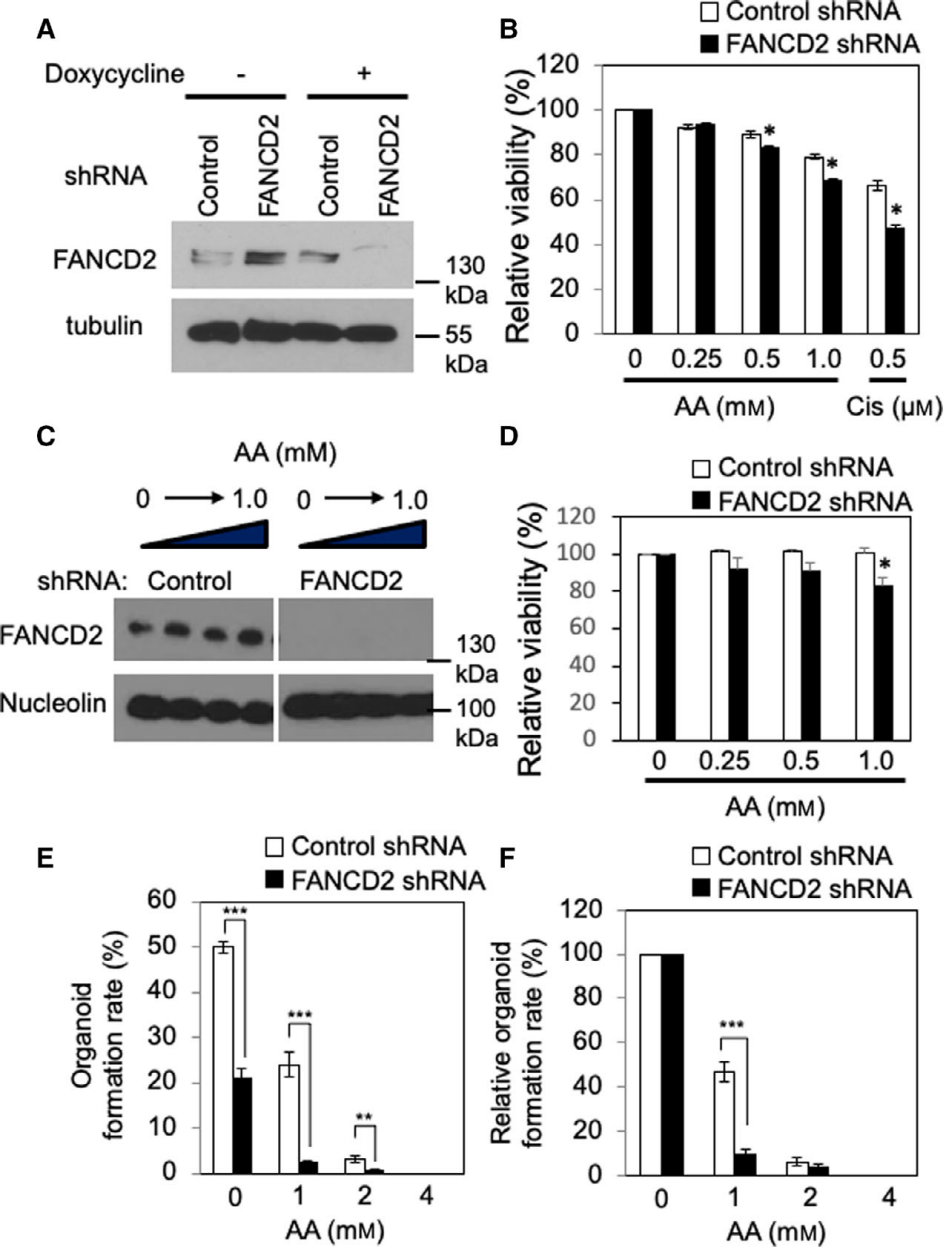
FANCD2 depletion exacerbates acetaldehyde‐induced loss of viability. (A) EPC2‐hTERT cells were depleted of FANCD2 via a doxycycline‐inducible shRNA lentiviral infection. Control and FANCD2‐depleted cells were lysed, and FANCD2 levels were assessed via western blot. Nucleolin was used as a loading control. (B) Control and FANCD2‐depleted EPC2‐hTERT cells were treated with up to 1 mm acetaldehyde (AA) for 48 h, and cell viability was assessed via trypan blue exclusion. Relative cell viability was calculated against viability of nontreated cells (0 mm AA) *n* = 3. Student's *t*‐test, *P < 0.05. Error bars indicate SEM. (C) TE11 cells constitutively expressing control or FANCD2 shRNAs were treated with indicated concentrations of acetaldehyde. Cells were selected by puromycin and lysed, and FANCD2 levels were monitored. (D) The cells used in *C* were also assessed for viability using trypan blue exclusion. Relative cell viability was calculated as described in B. (E) Control and FANCD2‐depleted TE11 cells were suspended in Matrigel and grown into 3D esophageal organoids. Cells were treated with up to 4 mm AA for the first 72 h of organoid formation, and then, acetaldehyde‐free media was added as organoids continued to grow for 10 days. Following organoid formation, organoids were photographed, and organoid formation rates (%) were calculated against the initial number of plated cells. (F) Relative organoid formation rates (%) were recalculated by normalizing organoid formation rates to those of untreated samples in both control and FANCD2 shRNA‐expressing cells. Student's *t*‐test; **P < 0.01 and ***P < 001. *n* = 3. Error bars indicate SEM.

The colocalization of FANCD2 and EdU signals in acetaldehyde‐treated esophageal keratinocytes (Fig. 4A,C) prompted us to investigate the role of the FA pathway in response to acetaldehyde‐induced replication stress. To address this, we depleted FANCD2 via shRNA from EPC2‐hTERT cells (Fig. 6A). Trypan blue exclusion assays revealed that FANCD2‐depleted cells were significantly more sensitive to acetaldehyde than control cells (Fig. 6B). In addition, siRNA‐mediated FANCD2 depletion resulted in elevated cellular sensitivity to acetaldehyde in EPC1‐hTERT and EPC2‐hTERT cells (Fig. S4). FANCD2‐depleted TE11 cells also showed increased sensitivity to acetaldehyde (Fig. 6C,D). Furthermore, to recapitulate human physiology, esophageal 3D organoids [25, 26, 43] were grown with TE11 cells and treated with acetaldehyde. Again, there was a reproducible reduction in the rate of organoid formation upon FANCD2 depletion in TE11 cells (Fig. 6E,F).

**Fig. 7:**
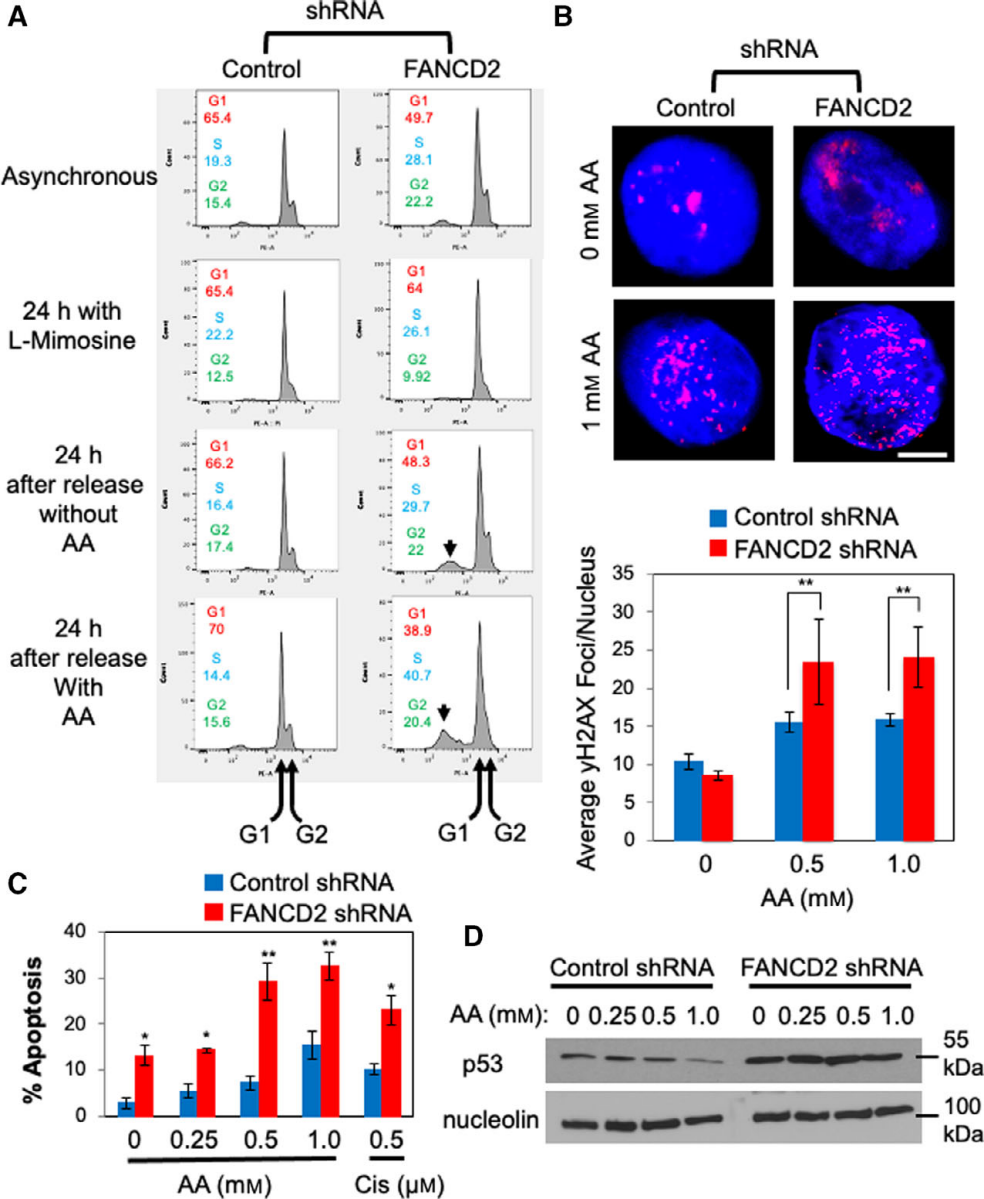
FANCD2 depletion alters DNA replication process and leads to genomic instability in esophageal keratinocytes. (A) EPC2‐hTERT cells constitutively expressing the indicated shRNA (asynchronous) were treated with l‐mimosine for 24 h to synchronized cells at G1/S and released into fresh medium in the presence or absence of acetaldehyde (AA). Twenty‐four hours after the release, cells were collected and processed for flow cytometry analysis using a LSRFortessa cell analyzer. Representative results of repeat experiments (*n* = 3) are shown. Average percentages of cell population in each cell cycle phase are indicated. (B) Control and FANCD2 shRNA‐expressing cells were treated with up to 1 mm AA for 24 h. Cells were then fixed and processed for immunofluorescence to detect yH2AX DNA damage foci. Scale bar, 10 µm. At least 50 cells were counted for each replicate. *n* = 3. Mann–Whitney *U*‐test, **P < 0.01. Error bars represent SEM. (C) Control and FANCD2‐depleted EPC2‐hTERT cells were treated with up to 1 mm AA for 48 h, and then, cells were stained with propidium iodide and Annexin V to assess early apoptotic cells. Ten thousand cells were counted for each sample. *n* = 3. Student's *t*‐test; *P < 0.05 and **P < 0.01. Error bars indicate SEM. (D) Control and FANCD2‐depleted cells were treated with up to 1 mm AA for 24 h, cells were lysed, and proteins were analyzed via western blot for p53 and nucleolin (loading control) expression.

We then assessed the effect of FANCD2 depletion on the DNA replication process. For this purpose, control and FANCD2 shRNA‐expressing EPC2‐hTERT cells were synchronized at the G1/S boundary with l‐mimosine and released into the cell cycle in the presence or absence of acetaldehyde for 24 h. As shown in Fig. 7A, l‐mimosine‐treated cells displayed a sharp G1 peak, while showing a reduced G2 peak in both control and FANCD2‐depleted cells. When cells were released into the cell cycle in the absence of acetaldehyde for 24 h, clear G2 peaks reappeared in both control and FANCD2‐depleted cells (Fig. 7A), indicating that cells are undergoing DNA replication and moving into G2 phase. Interestingly, FANCD2‐depleted cells had increased S‐phase population compared to control cells (Fig. 7A). Importantly, FANCD2‐depleted cells showed robust accumulation of S‐phase cells in the presence of acetaldehyde, whereas S‐phase population in control cells remained relatively low (Fig. 7A). Thus, FANCD2 is involved in limiting DNA replication stress to facilitate DNA replication in response to acetaldehyde.

It is possible that such acetaldehyde‐induced replication abnormalities can result in increased levels of DNA damage when FANCD2 is depleted. To test this possibility, we monitored DNA damage foci formation of FANCD2‐depleted EPC2‐hTERT cells. For this purpose, we monitored localization of γH2AX, a general DNA damage marker. When compared to control cells, γH2AX foci formation was highly elevated in FANCD2‐depleted cells in response to acetaldehyde (Fig. 7B). Thus, our results suggest that FANCD2 also limits the acetaldehyde‐induced DNA damage in esophageal cells.

We hypothesized that the elevated DNA damage in FANCD2‐depleted cells triggers apoptotic cell death. Indeed, cells with sub‐G1 DNA contents were elevated in FANCD2‐depleted cells (Fig. 7A, arrows), suggesting a role of FANCD2 in limiting apoptosis in response to acetaldehyde. Indeed, Annexin V/propidium iodide double staining of acetaldehyde‐treated cells revealed that acetaldehyde induced significantly increased levels of apoptosis in FANCD2‐depleted esophageal keratinocytes when compared to control cells (Fig. 7C). Furthermore, we observed FANCD2‐depleted EPC2‐hTERT cells have generally elevated levels of p53, the tumor suppressor responsible for DNA damage response (Fig. 7D), further confirming the role of FANCD2 in preventing accumulation of DNA damage in response to acetaldehyde in esophageal keratinocytes.

**Fig. 8:**
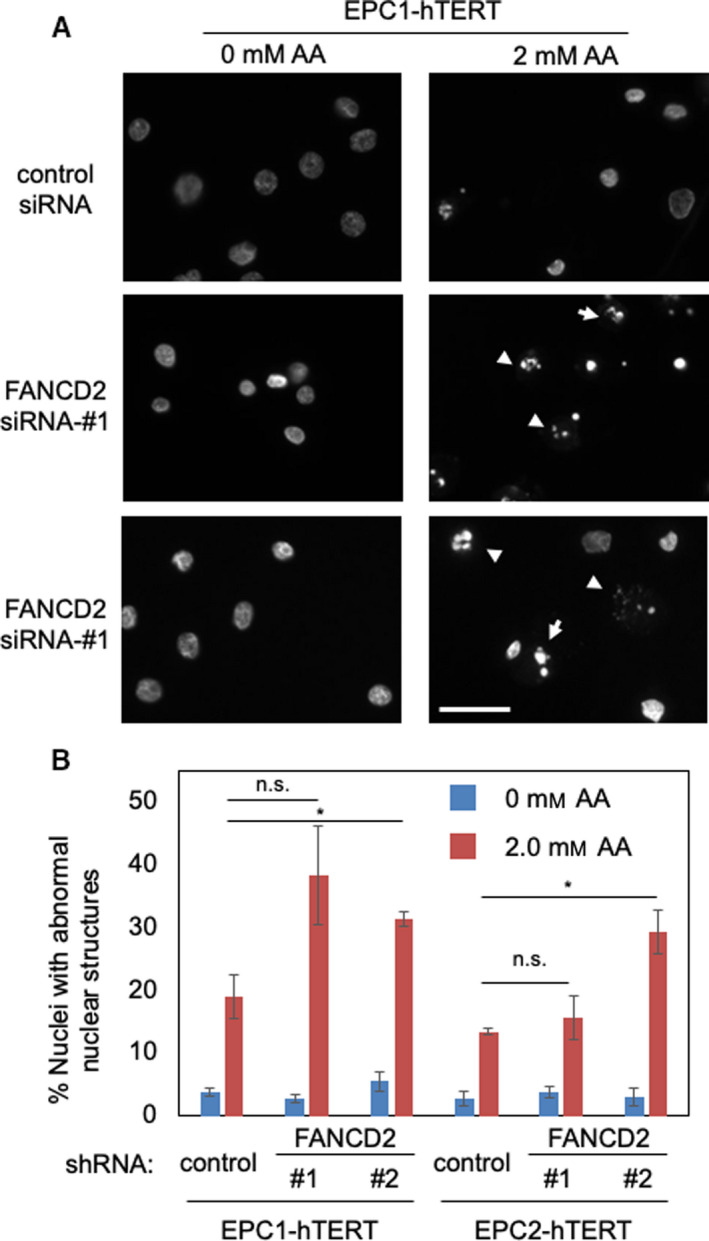
FANCD2 depletion results in formation of abnormal nuclear structures. (A) EPC1‐hTERT and EPC2‐hTERT cells were transfected with the indicated siRNA oligonucleotides. Two days after siRNA transfection, EPC1‐hTERT and EPC2‐hTERT cells were treated with or without 2 mm acetaldehyde (AA) for 3 days. Cells were then fixed and processed for DAPI staining. Arrows and arrowheads indicate micronuclei and apoptotic nuclei, respectively. Representative images are shown. (B) Percentages of cells with abnormal nuclear structures are shown. Scale bar, 100 µm. At least 100 cells were counted for each experiment. Error bars correspond to SEM. *n* = 3 for siRNA‐#1. *n* = 4 for siRNA‐#2. Student's *t*‐test; *P < 0.05 and n.s., not significant.

To further address the role of FANCD2 in preventing genomic instability, we examined the formation of abnormal nuclear structures. As shown in Fig. 8, acetaldehyde induced a significant increase in the appearance of abnormal nuclear structures in both EPC1‐hTERT and EPC2‐hTERT cells (Fig. 8, Fig. S5). These abnormal nuclear structures include micronuclei, nuclear fragmentation, and other aberrant structures that can be caused by multiple causes including chromosome segregation defects and/or apoptosis. Many of these abnormal nuclei appear to be derived from apoptotic cells, which are likely due to increased levels of DNA damage and genomic instability when FANCD2 is depleted. This result is also consistent with the elevated apoptosis shown in Fig. 7C. Importantly, FANCD2 siRNA treatment exacerbated the formation of abnormal nuclear structures in these cells (Fig. 8, Fig. S5). Thus, our results are consistent with the notion that FANCD2 limits acetaldehyde‐induced genomic instability.

## 4 Discussion

Several studies have used esophageal models to understand the role of acetaldehyde in inducing genomic instability. For example, loss of ALDH2 causes elevated levels of γH2AX and acetaldehyde‐derived *N* ^2^‐ethylidene‐2′‐deoxyguanosine in the mouse esophagus after ethanol administration [35, 44, 45, 46]. The *N* ^2^‐ethylidene‐2′‐deoxyguanosine level was also elevated in EPC2‐hTERT human esophageal keratinocytes in response to acetaldehyde [35]. These results suggest that acetaldehyde causes DNA damage in the esophagus, although the nature of acetaldehyde‐induced DNA damage was not investigated in these studies.

In this report, we have characterized how acetaldehyde alters the DNA replication process and causes genomic instability in esophageal keratinocytes. Interestingly, in CHO cells, acetaldehyde causes G2/M cell cycle arrest and increased levels of ssDNA, suggesting that acetaldehyde‐induced DNA adducts interferes with the DNA replication process [47]. In human DLD1 colorectal adenocarcinoma cells, DNA combing analysis revealed that acetaldehyde causes replication stress especially in the absence of BRCA2 [48]. Consistently, we have found that acetaldehyde causes G2/M cell cycle arrest via the ATR‐Chk1‐dependent DNA damage response in esophageal keratinocytes (Fig. 1, Fig. S3), suggesting the role of ATR‐Chk1 in limiting DNA replication abnormalities. In addition, our EdU incorporation assays have revealed that cells accumulate in early S phases, further supporting a notion that acetaldehyde‐induced DNA adducts interfere with DNA replication stress in esophageal keratinocytes (Fig. 4).

Our studies have demonstrated the role of replication fork protection in the tolerance to acetaldehyde‐induced genomic instability. When Timeless, a subunit of the replication fork protection complex [42], is depleted, cells shows elevated DNA damage and slow growth phenotypes (Fig. 5). This is consistent with our␣previous finding that the fission yeast Timeless homolog is necessary to stabilize replication forks at␣acetaldehyde‐dependent DNA adducts [8]. Such acetaldehyde‐induced DNA adducts may include ICLs and DPCs, which must be removed by DNA repair mechanisms. Although mechanisms of DPC repair are less understood [12, 49], it is known that ICL repair requires the FA DNA repair pathway [4, 5]. Consistently, loss of the FA pathway is reported to cause acetaldehyde sensitivity and genomic instability in response to acetaldehyde [10, 11, 15, 16, 17, 18, 19]. Importantly, we have demonstrated the importance of the FA pathway in limiting replication stress and genomic instability in esophageal keratinocytes, a model to understand mechanisms of esophageal carcinogenesis. After acetaldehyde treatment, FANCD2 is monoubiquitinated and forms DNA damage foci at sites of DNA synthesis (Fig. 3). FANCD2 depletion causes an exacerbation of acetaldehyde‐induced DNA damage, resulting in increased γH2AX foci and decreased cell viability in both 2D and 3D cultures (Figs 6 and 7B, Fig. S4); thus, the disruption of the FA pathway prevents efficient DNA repair in esophageal keratinocytes. In addition, we have shown that FANCD2‐depleted cells displayed impaired cell cycle progression when cells were released from G1/S into the cell cycle in the presence of acetaldehyde (Fig. 7A). Furthermore, FANCD2 limits acetaldehyde‐induced genomic instability in esophageal keratinocytes, as FANCD2 depletion results in an increase of cells with abnormal nuclear structures (Fig. 8). These results highlight the importance of the FA pathway in the resolution of acetaldehyde‐induced DNA damage that may contribute to the development of esophageal cancer.

The FA pathway plays a critical role in the maintenance of HSCs [11, 16, 50, 51, 52]. In the absence of FANCD2, cells accumulate DNA damage, which results in hyperactivation of p53. This, in turn, leads to p21‐dependent cell cycle arrest in hematopoietic stem and progenitor cells (HSPCs), contributing to attrition of those cells in FA patients [50]. Importantly, loss of p53 rescued these HSPC defects, suggesting a potential clinical intervention for FA patients [50]. Consistently, in acetaldehyde‐treated esophageal keratinocytes, p53 is highly stabilized and that apoptosis is activated in the absence of FANCD2 (Fig. 7). In this situation, p53 may play an important role in preventing damaged FA‐deficient cells from passing down incorrect genetic information to the next generation. Therefore, p53 inactivation may cause increased genetic instability and/or mutation rate in esophageal cells, leading to malignant transformation. This notion is supported by the fact that p53 mutations are the most frequent genetic alteration in ESCC (approximately 50%), the most common type of esophageal cancer [53]. In addition, ESCC in FA patients is often associated with infection by human papillomaviruses, which contain E6 protein to suppress p53 activity [54, 55, 56]. Furthermore, p53 dysfunction promotes leukemogenesis in FA patients [57, 58]. Therefore, further investigation is warranted to investigate how p53 and FA proteins collectively affect acetaldehyde‐induced DNA damage and repair processes in esophageal carcinogenesis.

## 5 Conclusions

In summary, our work has provided important insights regarding how acetaldehyde induces genomic instability in esophageal keratinocytes. We demonstrated that acetaldehyde causes DNA replication stress, leading to activation of the FA pathway in esophageal keratinocytes. We also provided evidence that the FA pathway limits acetaldehyde‐induced replication stress and genomic instability. These studies will serve as a guiding investigation of genome maintenance mechanisms in FA‐ and alcohol‐associated esophageal carcinogenesis, building a platform for new translational applications for prevention and therapy of ESCC.

## Conflict of interest

The authors declare no conflict of interest.

## Author contributions

JDP and EN conceived and designed experiments. JDP, CN, BL, AT, MO, SS, and KT performed experiments. JDP, CN, BL, AT, SS, HN, and EN analyzed the data. JDP, CN, BL, AT, MO, SS, KT, HN, and EN contributed to reagents, materials, and analysis tools. JDP and EN wrote the paper.

## Supporting information


**Fig.␣S1.** Acetaldehyde treatment scheme.
**Fig.␣S2.** Effect of acetaldehyde exposure on TE11 cells.
**Fig.␣S3.** Acetaldehyde induces Chk1 phosphorylation in esophageal keratinocytes.
**Fig.␣S4.** siRNA‐mediated FANCD2 depletion results in an increased cellular sensitivity to acetaldehyde in esophageal keratinocytes.
**Fig.␣S5.** Abnormal nuclear structures in acetaldehyde‐treated EPC2‐hTERT cells.

## Data Availability

The data that support the findings of this study are available from the corresponding author (en34@drexel.edu) upon reasonable request.
