# Supplementary material for: FANCD2 limits acetaldehyde‐induced genomic instability during DNA replication in esophageal keratinocytes
Source: Mol Oncol. 2021 Aug 8;15(11):3109–24. doi: 10.1002/1878-0261.13072 (PMC8564632; doi:10.1002/1878-0261.13072)
Supplement: Supplementary file 1 — Fig.␣S1. Acetaldehyde treatment scheme. Fig.␣S2. Effect of acetaldehyde exposure on TE11 cells. Fig.␣S3. Acetaldehyde induces Chk1 phosphorylation in esophageal keratinocytes. Fig.␣S4. siRNA‐mediated FANCD2 depletion results in an increased cellular sensitivity to acetaldehyde in esophageal keratinocytes. Fig.␣S5. Abnormal nuclear structures in acetaldehyde‐treated EPC2‐hTERT cells. [file MOL2-15-3109-s001.pdf]

Supplementary Figure S1

Acetaldehyde Treatment Scheme

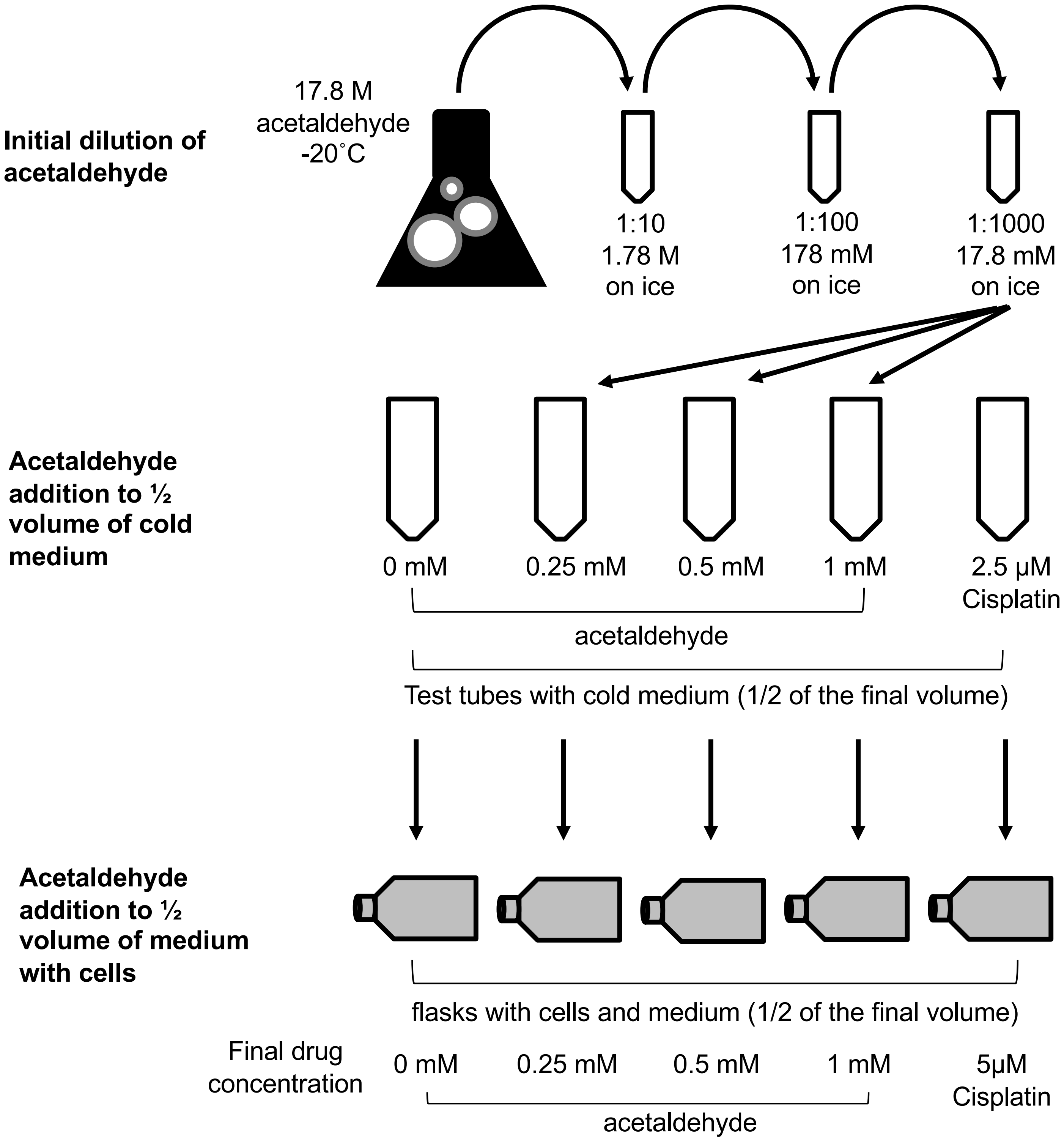

Supplementary Figure S2

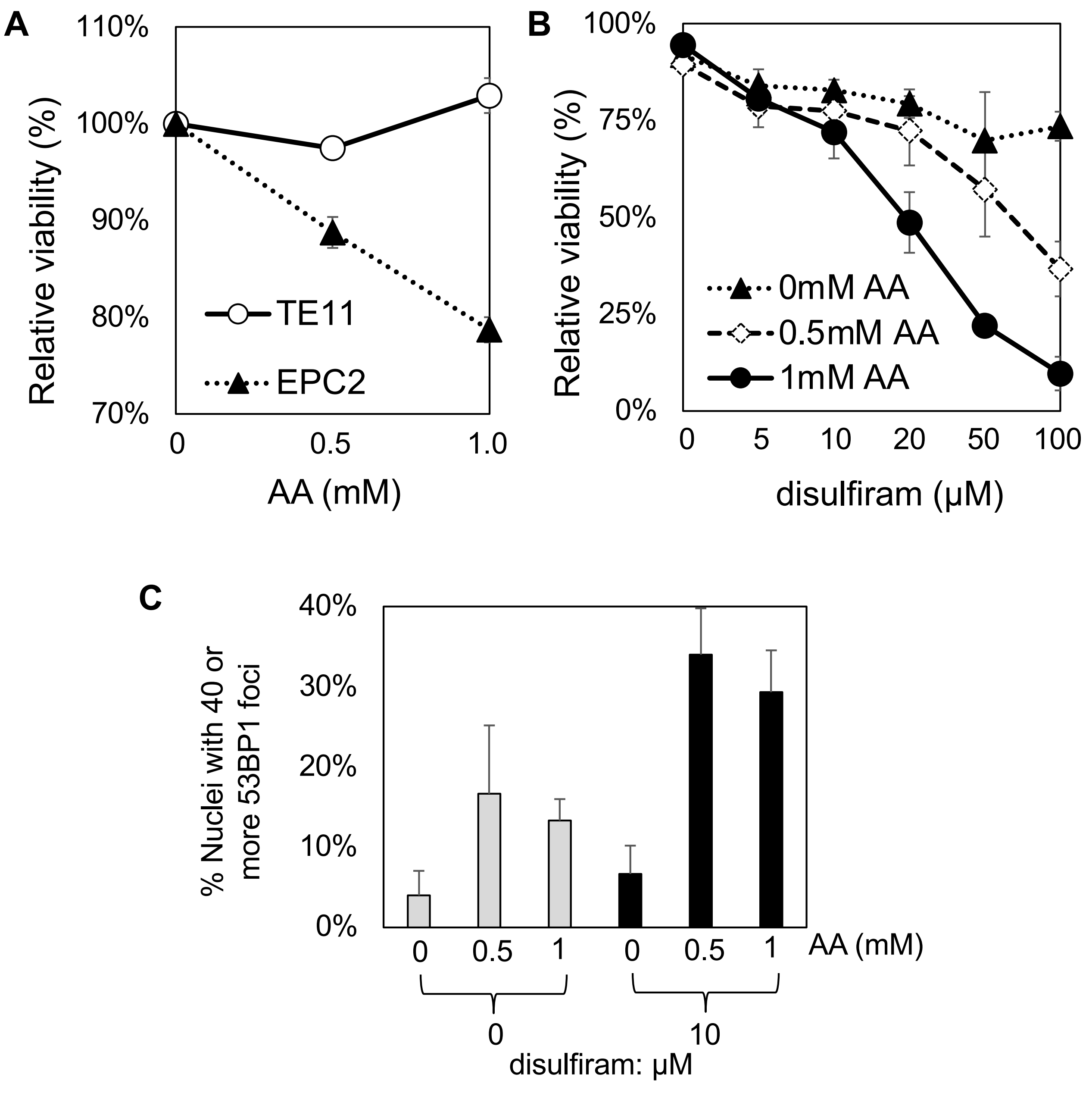

Supplementary Figure S3

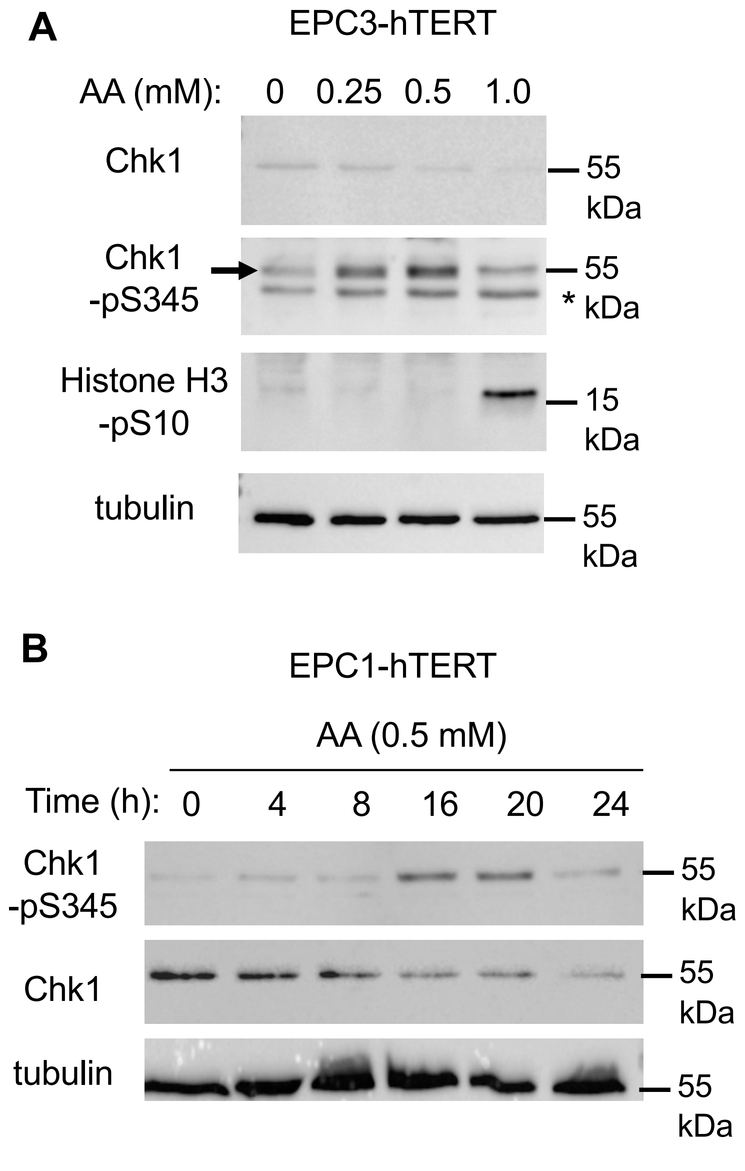

Supplementary Figure S4

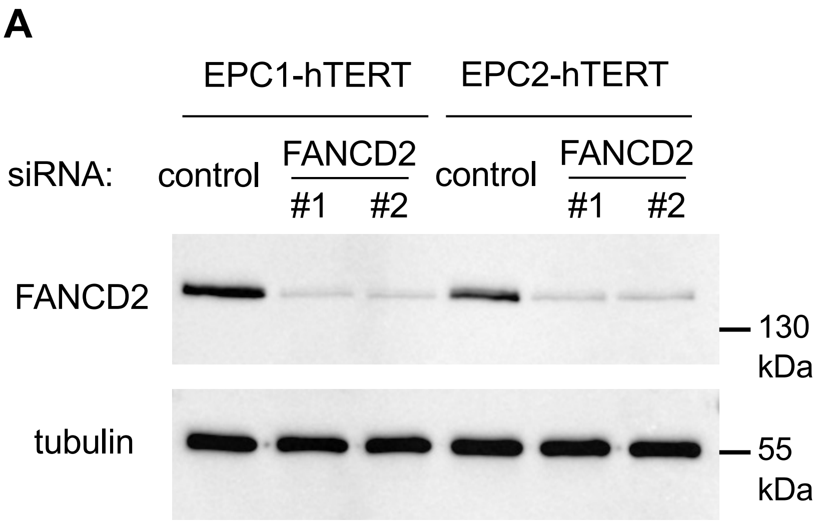

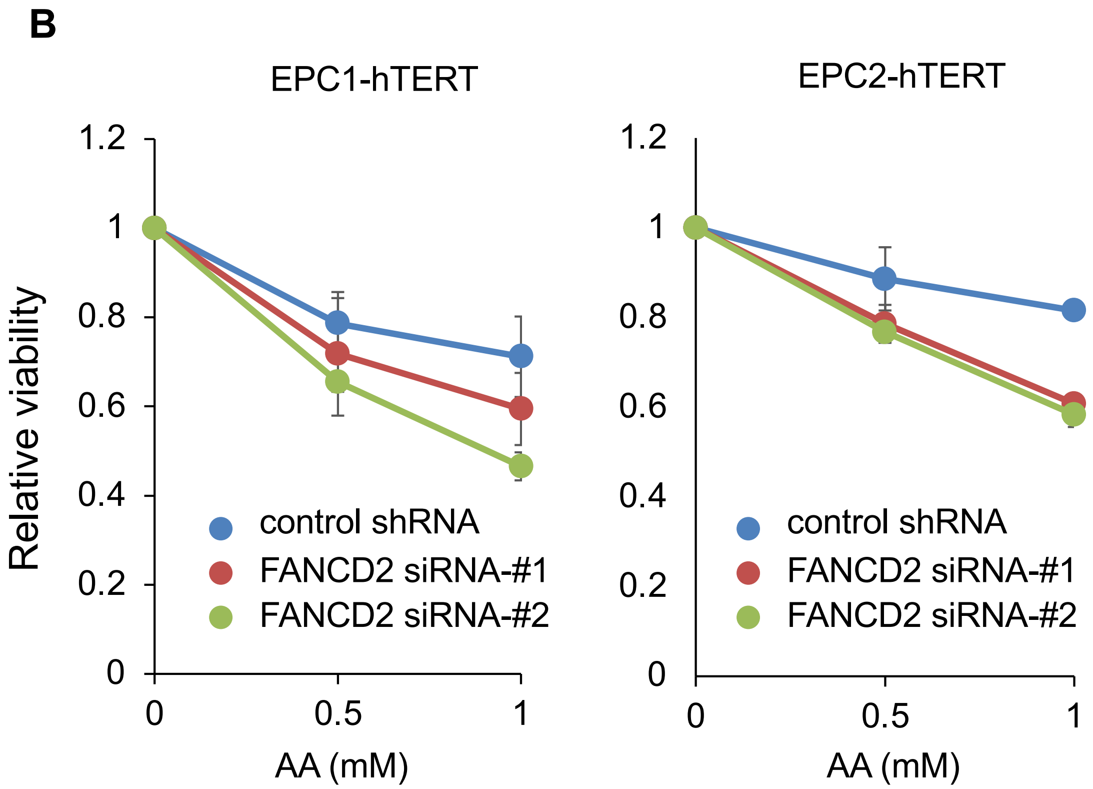

Supplementary Figure S5

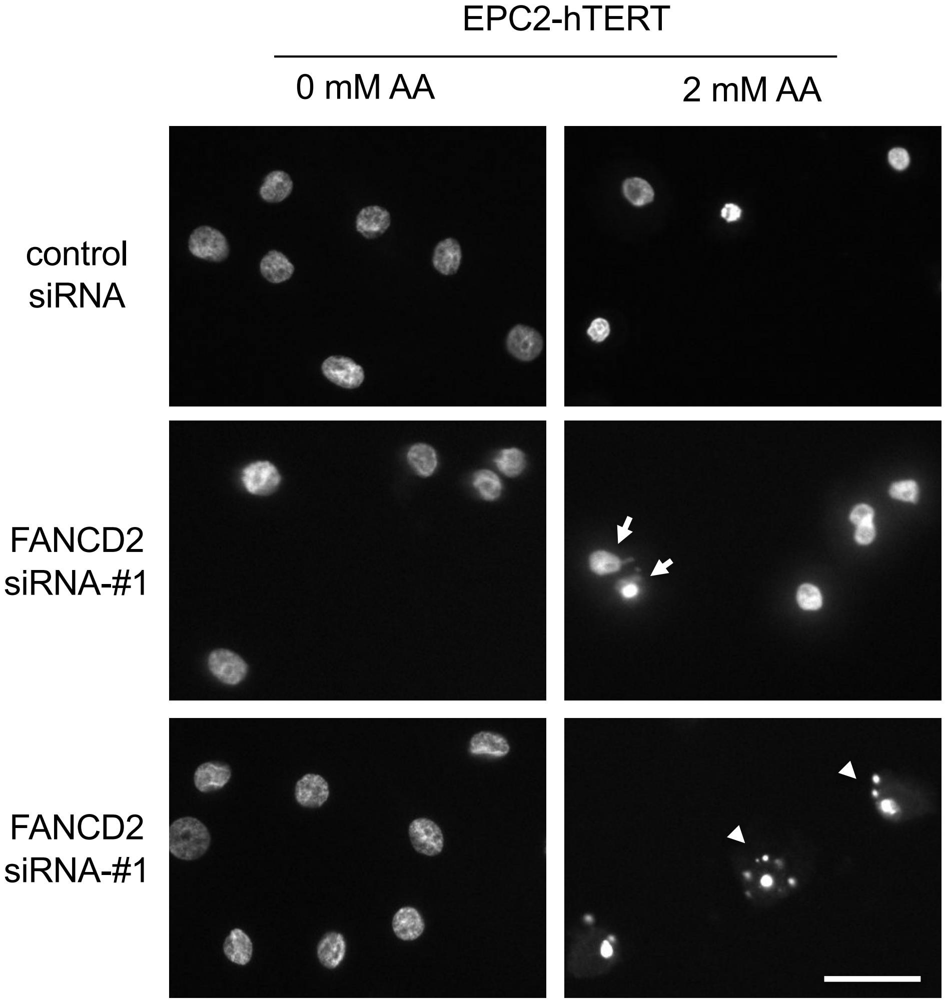

Supplementary Figure Legends

Supplementary Figure S1: Acetaldehyde treatment scheme. While on ice, acetaldehyde was diluted ten-fold until a 1:1000 dilution was achieved. Next, the diluted acetaldehyde was quickly added to test tubes containing cold growth medium to reach twice the final acetaldehyde concentration. The acetaldehyde-containing medium was added at 1:1 ratio to culture flasks containing cells with medium, and the lid was tightened to minimize acetaldehyde evaporation. Non-treated and cisplatin-treated cells were processed in the same conditions. See Materials and Methods for details.

Supplementary Figure S2: Effect of acetaldehyde exposure on TE11 cells. A) EPC2-hTERT and TE11 cells were treated with the indicated concentration of acetaldehyde (AA) for 48 hours. Cells were then collected and counted to determine the cell viability via trypan blue staining. The cell viability was calculated by normalizing the number of viable cells to that of non-treated cells. **B)** TE11 cells were treated with the indicated concentration of disulfiram or DMSO (vehicle control) for 30 minutes, and then exposed to the indicated amounts of acetaldehyde (AA) for 48 hours. After treatment, cells were collected and counted to determine cell viability via trypan blue staining. Error bars correspond to SEM obtained from three independent experiments. **C)** TE11 cells were incubated with or without disulfiram for 30 minutes prior to acetaldehyde (AA) treatment (24 hours) and processed for 53BP1 immunofluorescence microscopy. Quantification of 53BP1 foci was expressed as percentage of nuclei with over 40 foci in each treatment. Error bars correspond to SEM obtained from three independent experiments.

Supplementary Figure S3: Acetaldehyde induces Chk1 phosphorylation in esophageal keratinocytes. A) EPC3-hTERT cells were treated with up to 1 mM acetaldehyde (AA) for 24 hours. Cell lysates were prepared and probed for the indicated proteins. Arrows indicate Chk1 phosphorylation on S345. Asterisk indicates non-specific bands. Tubulin was used as a loading control. **B)** EPC1-hTERT cells were treated with 0.5 mM acetaldehyde (AA) for the indicated times and processed for immunoblot analyses with the indicated antibodies. Representative results of repeat experiments are shown.

Supplementary Figure S4: siRNA-mediated FANCD2 depletion results in an increased cellular sensitivity to acetaldehyde in esophageal keratinocytes. A) EPC1-hTERT and EPC2-hTERT cells were transfected with the indicated siRNA oligonucleotides. Cells were lysed, and FANCD2 levels were assessed via Western blot. Tubulin was used as a loading control. **(B)** Control and FANCD2-depleted EPC1-hTERT cells were treated with up to 2 mM acetaldehyde (AA) for 72 hours, and cell viability was measured via WST-8 assay. Relative cell viability was calculated against viability of non-treated cells (0 mM acetaldehyde, AA). Error bars correspond to SEM obtained from triplicates. Representative results are shown.

Supplementary Figure S5: Abnormal nuclear structures in acetaldehyde-treated EPC2-hTERT cells. EPC2-hTERT cells were transfected with the indicated siRNA oligonucleotides and treated with or without acetaldehyde (AA) for 3 days. Cells were then fixed and processed for DAPI staining. Arrows and arrowheads indicate

micronuclei and apoptotic nuclei, respectively. Representative images are shown. Scale bar, 100 μm. Quantification of this result is shown in Figure 8A.
